# Effects of Herbal Essential Oil Mixture as a Dietary Supplement on Egg Production in Quail

**DOI:** 10.1155/2014/573470

**Published:** 2014-01-23

**Authors:** Metin Çabuk, Serdar Eratak, Ahmet Alçicek, Mehmet Bozkurt

**Affiliations:** ^1^Department of Poultry Science, Vocational School of Celal Bayar University, Akhisar, 45210 Manisa, Turkey; ^2^Department of Animal Science, Agricultural Faculty of Ege University, Bornova, 35100 Izmir, Turkey; ^3^Poultry Research Institute, Erbeyli, 09600 Aydın, Turkey

## Abstract

One hundred and eighty 7-week-old laying quail were fed various diets over a 12-week period. The diets included a control diet (without essential oil mixture (EOM) or antibiotics (ANTs)), a basal diet including EOM (24 mg/kg feed), and a basal diet including an ANT (avilamycin, 10 mg/kg feed). Each treatment comprised 4 replications with 4 cages (15 quail per cage), amounting to 60 quail per treatment group. Diets (in mash form) and water were provided for *ad libitum* consumption. EOM consisted of 6 different essential oils derived from the following herbs: oregano (*Origanum* sp.), laurel leaf (*Laurus nobilis* L.), sage leaf (*Salvia triloba* L.), myrtle leaf (*Myrtus communis*), fennel seeds (*Foeniculum vulgare*), and citrus peel (*Citrus* sp.). In comparison with the control diet, adding supplements such as EOM and ANTs to the basal diet increased egg production in quail (*P* < 0.001). However, egg production was similar between EOM and ANT treatment groups. Moreover, there were no differences between the treatment groups with regard to egg weight. Feed intake was not affected by EOM or ANT supplementation, whereas feed conversion ratio was significantly improved by EOM and ANT supplementation. Thus, we concluded that EOM has beneficial effects as a dietary supplement on egg production and feed conversion ratio.

## 1. Introduction

For several years, antibiotics (ANTs) have been extensively used worldwide as growth promoters in animal feeds, particularly in poultry and pig production. However, objections to the use of growth-promoting ANTs are increasing as consumers fear that their use may lead to the emergence of ANT-resistant bacteria that are harmful to humans. As a result, in several countries, efforts have been made to ban the use of all types of growth-promoting ANTs in animal feeds [1, 2]. These trends have created an industrial demand for ANT replacements such as herbal essential oils, probiotics, prebiotics, and organic acid botanicals [3, 4]. Essential oils help in the colonization and maintenance of balanced levels of beneficial microbial populations within the gastrointestinal system [5, 6]. In addition to their antimicrobial properties [7], essential oils also exhibit antioxidant [8, 9] and antifungal [9, 10] properties. Therefore, one alternative to antimicrobial feed additives is essential oils derived from herbs and spices. The beneficial effects of essential oils on animal nutrition include appetite stimulation as well as improvement of endogenous digestive enzyme secretion and immune response activation [8, 11, 12]. Some essential oils also exert antiheat stress effects [13, 14]. Several studies have shown that herbal essential oils exhibit antibacterial activities in the gut lumen and act as growth promoters in early life of pigs and broiler chickens [15, 16]. This practice is currently receiving considerable attention, particularly in broiler chickens [17, 18]. The aim of the present study was to investigate the effects of essential oil mixture (EOM) as a dietary supplement on egg production, egg weight, feed conversion ratio (FCR), and feed consumption in laying quail.

## 2. Materials and Methods

One hundred and eighty 7-week-old laying quail (*Coturnix coturnix japonica*) were fed various diets over a 12-week period. The diets included a control diet (without EOM or ANTs), a basal diet including EOM (24 mg/kg feed), and a basal diet including an ANT (avilamycin, 10 mg/kg feed). Each treatment comprised 4 replications with 4 cages (15 quail per cage), amounting to 60 quail per treatment group. EOM (Heryumix Herba Ltd., Co., İzmir, Turkey) included carvacrol, thymol, 1:8-cineole, *p*-cymene, and limonene as active components and 6 different essential oils derived from the following herbs: oregano (*Origanum* sp.), laurel leaf (*Laurus nobilis* L.), sage leaf (*Salvia triloba* L.), myrtle leaf (*Myrtus communis*), fennel seeds (*Foeniculum vulgare*), and citrus peel (*Citrus* sp.). EOM used 976 g of zeolite as a carrier for 24 g of essential oil. An ANT preparation containing 10,000 mg of avilamycin per kg of premix (Kavilamycin, Kartal Kimya-BASF, Gebze, Turkey) was also investigated as a potential dietary supplement. EOM and ANT premixes were added as supplements to the basal diet. One kg of supplement per 1,000 kg of feed was added in place of sawdust, which is typically included in basal diets as inert filler. All diets were isocaloric and isonitrogenous. Diets (in mash form) and water were provided for *ad libitum* consumption. The ingredients and chemical composition of the basal diets are presented in Table 1.

A photoperiod of 17 h/day was maintained. Egg production was recorded daily. During the laying period, a random sample of 10 eggs/replicate was collected on 2 consecutive days per week for determining egg weight. Feed intake was recorded on a weekly basis. FCR was calculated as the ratio of g of feed consumed per g of egg weight. The magnitude of production variables such as feed intake and egg production was adjusted for mortality rates. Mortality was recorded daily. Albumen quality was measured in terms of Haugh units (HUs) calculated from the weight of the egg and the height of the albumen [19]. HU values were calculated for each egg using the following formula: HU = 100log⁡(*H* − 1.7*W* ^0.37^ + 7.6), where *H* is the observed height of the albumen (mm) and *W* is the weight of the egg (g). The yolk colour was measured using the Roche yolk colour fan [20]. Data were analysed with the general linear model procedure of SAS [21]. Differences were considered statistically significant at *P* < 0.05.

## 3. Results and Discussion

The effects of dietary supplements on egg production, feed intake, FCR, and egg weight are shown in Table 2.

Supplementing the basal diet with EOM and ANT significantly increased egg production and improved FCR in comparison with the control diet. However, egg production and FCR were similar between EOM and ANT treatment groups. A similar result was observed by Ma et al. [14] who found that a diet supplemented with herbal medicine (*Ligustrum lucidum* and *Schisandra chinensis*) significantly improved egg production and FCR in laying hens. In addition, a recent study [22] found that in comparison with a control diet, a diet supplemented with EOM significantly improved egg production and FCR in laying hens. Our findings are in agreement with those of Bölükbaşı et al. [23] who reported that dietary supplementation with mixed essential oils and thyme oil improved the egg production rate by 5.43% and 6.20%, respectively. However, contrasting results have been reported by Bozkurt et al. [24] who indicated that a diet supplemented with EOM had no beneficial effects on egg production or FCR in laying hens. Similarly, addition of oregano oil [25] and thyme oil [23] to a layer diet had no significant effects on FCR. In our study, addition of EOM and ANT to the layer diet had no significant effects on daily feed intake of laying quail. Although our data is in accordance with that of several preliminary studies [22, 24–27], it contradicts some other studies that examined reduced feed intake. Egg weight did not differ between the different treatment groups. Our findings are in agreement with those of Bozkurt et al. [24] who studied the effects of EOM in laying hens. Similar results were reported by Çabuk et al. [22] who indicated that a diet supplemented with EOM had no beneficial effect on egg weight. In contrast to our results, Bölükbaşı et al. [28] observed that the addition of essential oils to a layer diet increased egg weight. The effects of EOM and ANT on egg quality characteristics and body weight are shown in Table 3.

Egg quality characteristics, including HU, shell thickness, shell weight, yolk colour, yolk weight, albumen index, and yolk index, were not significantly affected by supplementing the diet with EOM or ANT in laying quail. These results are in agreement with those of Bozkurt et al. [24] who examined the effects of EOM on shell weight, shell thickness, and albumen index. In the present study, the final body weights of quail did not differ significantly between the different treatment groups. Similarly, previous studies also reported no beneficial effects on body weight of hens that were fed diets supplemented with oregano oil or EOM [25, 29]. In contrast to our results, Çabuk et al. [22] observed that supplementing a layer diet with essential oils increased the body weight in laying hens.

## 4. Conclusions

The results of this study indicate that EOM has beneficial effects as a dietary supplement on egg production and FCR. These results also suggest that supplementing diets with herbal EOM of natural origin is a viable alternative to growth-promoting ANTs in poultry nutrition.

## Figures and Tables

**Table 1:** Ingredient and chemical composition of the basal experimental layer quail diet (as fed).

Ingredient, %	
Corn	52.76
Soybean meal (%44 CP)	25.78
Full fat soybean	8.00
Sunflower oil	2.47
Ground limestone	8.29
Dicalcium phosphate	1.29
Vitamin-mineral premix*	0.25
DL-methionine	0.71
Lysine	0.10
Salt	0.25
Sawdust**	0.10
Total	**100.00**

Calculated composition, %	

Dry matter	89.42
Crude protein	19.00
Crude fiber	2.77
Crude ash	12.94
Ether extract	6.02
Met + Cys	0.75
Lysine	1.14
Calcium	3.50
ME, kcal/kg	2900
Total phosphorus	0.55
Available phosphorus	0.41

*Supplied per kg of diet: vitamin A 12 000 IU; vitamin D3 3 000 IU; vitamin E 30 mg; vitamin K3 5 mg; vitamin B1, 3 mg; vitamin B2 12 mg; vitamin B6 4 mg; vitamin B12 0.03 mg; nicotine amid 55 mg; calcium-D-pantothenate 15 mg; folic acid 2 mg; D-biotin 0.250 mg; choline chloride 600 mg; Mn 80 mg; Fe 40 mg; Zn 60 mg; Cu 5 mg; I 0.4 mg; Co 0.1 mg; Se 0.15 mg. **Sawdust was substituted by antibiotic or essential oil mixture preparations.

**Table 2:** The effect of the dietary inclusion of essential oil mixture or an antibiotic on egg production, feed conversion ratio, and feed intake of quail.

Parameter	Control	EOM	ANT (avilamycin)	Pooled SEM	*P*
Egg production, %	86.52^b^	89.44^a^	88.34^a^	0.43	0.0010
Egg weight, g	11.80	11.69	11.77	0.52	0.2685
Feed intake, g/quail	29.24	29.14	29.13	0.17	0.8900
Feed conversion ratio, g fed/g egg	2.88^a^	2.81^b^	2.81^b^	0.02	0.0544

^a, b^Means within row with no common superscripts differ significantly (*P* < 0.05).

SEM: standard error of the mean.

**Table 3:** The effect of the dietary inclusion of essential oil mixture or an antibiotic on body weight, Haugh unit, egg yolk color, egg yolk weight, shell weight, and albumen index.

Parameter	Control	EOM	ANT (avilamycin)	Pooled SEM	*P*
Haugh unit	83.87	84.04	84.47	0.481	0.6636
Shell thickness, mm	0.211	0.208	0.206	0.011	0.0812
Shell weight, g	0.971	0.969	0.995	0.010	0.1185
Egg yolk color	7.64	7.62	7.67	0.154	0.9820
Egg yolk weight, g	3.65	3.59	3.68	0.032	0.1252
Albumen index	8.85	8.82	8.60	0.198	0.6395
Yolk index	46.19^b^	47.66^a^	46.32^b^	0.367	0.0084
Initial body weight, g	229.25	233.69	232.24	2.65	0.4853
Final body weight, g	242.59	242.02	243.11	3.35	0.9748

^a, b^Means within row with no common superscripts differ significantly (*P* < 0.05).

SEM: Standard error of the mean.
